# BDNF genetic variants and methylation: effects on cognition in major depressive disorder

**DOI:** 10.1038/s41398-019-0601-8

**Published:** 2019-10-21

**Authors:** Alex Ferrer, Javier Labad, Neus Salvat-Pujol, Marta Barrachina, Javier Costas, Mikel Urretavizcaya, Aida de Arriba-Arnau, José M. Crespo, Carles Soriano-Mas, Ángel Carracedo, José M. Menchón, Virginia Soria

**Affiliations:** 1 Department of Psychiatry, Bellvitge University Hospital, Bellvitge Biomedical Research Institute (IDIBELL), Neurosciences Group - Psychiatry and Mental Health, Barcelona, Spain; 2 Department of Mental Health, ParcTaulí Hospital Universitari, Institut d’Investigació i Innovació Parc Taulí (I3PT), Sabadell, Spain; 3 Department of Psychiatry and Legal Medicine, Universitat Autònoma de Barcelona, Barcelona, Spain; 4 Centro de Investigación Biomédica en Red de Salud Mental (CIBERSAM), Carlos III Health Institute, Madrid, Spain; 5 Neuropathology Group, Bellvitge University Hospital, Bellvitge Biomedical Research Institute (IDIBELL), L’Hospitalet de Llobregat, Barcelona, Spain; 6 Centro de Investigación Biomédica en Red de Enfermedades Neurodegenerativas (CIBERNED), Carlos III Health Institute, Madrid, Spain; 7 Grupo de Xenética Psiquiátrica, Instituto de Investigación Sanitaria de Santiago, Complexo Hospitalario Universitario de Santiago de Compostela, Servizo Galego de Saúde, Santiago de Compostela, Spain; 8 Department of Clinical Sciences, School of Medicine, Universitat de Barcelona, Barcelona, Spain; 9 Department of Psychobiology and Methodology of Health Sciences, Universitat Autònoma de Barcelona, Barcelona, Spain; 10 Fundación Pública Galega de Medicina Xenómica, Servicio Galego de Saúde, Instituto de Investigación Sanitaria de Santiago de Compostela, Santiago de Compostela, Spain; 11 Grupo de Medicina Xenómica, Universidade de Santiago de Compostela, Centro Nacional de Genotipado - Instituto Carlos III, Santiago de Compostela, Spain; 12 Centro de Investigación Biomédica en Red de Enfermedades Raras (CIBERER), Carlos III Health Institute, Madrid, Spain

**Keywords:** Prognostic markers, Clinical genetics

## Abstract

Brain-derived neurotrophic factor (*BDNF*) gene regulation has been linked to the pathophysiology of major depressive disorder (MDD). MDD patients show cognitive deficits, and altered *BDNF* regulation has a relevant role in neurocognitive functions. Our goal was to explore the association between *BDNF* genetic and epigenetic variations with neurocognitive performance in a group of MDD patients and healthy controls considering possible modulating factors. The sample included 134 subjects, 64 MDD patients, and 70 healthy controls. Clinical data, childhood maltreatment, and neurocognitive performance were assessed in all participants. Eleven single nucleotide polymorphisms (SNPs) and two promoter regions in the *BDNF* gene were selected for genotype and methylation analysis. The role of interactions between *BDNF* genetic and epigenetic variations with MDD diagnosis, sex, and Childhood Trauma Questionnaire (CTQ) scores was also explored. We observed significant associations between neurocognitive performance and two *BDNF* SNPs (rs908867 and rs925946), an effect that was significantly mediated by methylation values at specific promoter I sites. We identified significant associations between neurocognitive results and methylation status as well as its interactions with MDD diagnosis, sex, and CTQ scores. Our results support the hypothesis that *BDNF* gene SNPs and methylation status, as well as their interactions with modulating factors, can influence cognition. Further studies are required to confirm the effect of *BDNF* variations and cognitive function in larger samples.

## Introduction

Major depressive disorder (MDD) is a complex and highly prevalent psychiatric disorder with a high impact on quality of life and negative effects on mood, behavior, and cognition^1^. Cognitive dysfunction in MDD patients is a source of disability involving deficits in visual, verbal and working memory, attention, executive function, and processing speed^1^.

Several neurobiological mechanisms have been involved in the pathogenesis of MDD and related cognitive phenotypes, such as the neurotrophic signaling pathway. The brain-derived neurotrophic factor (*BDNF*) gene codes for a neurotrophin that is highly expressed in the central nervous system (CNS), mediates a variety of neuroplasticity processes, and has relevant influences on cognition and behavior^2^. BDNF is produced in the CNS and released to the extracellular matrix where it can interact with its receptors, tropomyosin-related kinase receptor B (TrkB) and p75 neurotrophin receptor (p75NTR), which mediate its effects^3^. Blood BDNF concentrations, that have been observed to correlate with BDNF expression levels in the brain^4^, are lower in MDD patients than in healthy controls^5^.

Research on the role of *BDNF* single-nucleotide polymorphisms (SNPs) in risk for MDD has been centered on rs6265^2^. This SNP, located in exon IX, results in a valine-methionine change in the pro-region of the pro-BDNF protein, which affects *BDNF* regulation. Overall, inconclusive results have been found, with positive data showing an association between the minor allele “A” and MDD^6,7^ and a lack of robust association in a recent meta-analysis^8^. The inconsistency of these results might be explained by methodological differences, heterogeneity of MDD clinical subtypes, the influence of modulating environmental factors and/or by epigenetic mechanisms. For instance, previous studies suggest that the Met allele of rs6265 moderates the relationship between stressful life events in childhood and depression^9,10^. In addition, rs6265 has been also associated with brain volumes^11^, presence of psychosis and suicidal behavior^12^, tendency to chronicity^13^ and treatment response in melancholic depression^14^.

Since *BDNF* is involved in processes such as neuronal survival, neurogenesis and long-term potentiation^15^, animal studies have reported a relevant role for rs6265 in motor learning and long-term memory^16^, which could be related to changes in hippocampal synaptic plasticity via downregulation of 5-HT3a receptors in rs6265Met carriers^17^. Human clinical studies have also reported significant associations between the *BDNF* genotype and cognitive performance^18,19^, although the results are often inconsistent and meta-analyses have been negative^20^.

Genome-wide association studies are beginning to identify associations of *BDNF* with several behavioral and cognitive traits^21^, such as smoking initiation^22^, educational attainment^23^, highest math class taken^23^, and the “worry” genetic-defined subcluster of neuroticism^24^.

DNA methylation is an epigenetic mechanism associated with gene silencing, although some evidence suggests that it may also be associated with active gene transcription^25^. Research on methylation patterns in *BDNF* of MDD patients has focused on promoter regions I and IV. Although most research has found that MDD patients show higher methylation levels at promoter I, some studies have reported an inverse association in severely depressed patients^26^. Research on methylation of promoter IV has also shown increased methylation levels in patients with depression when compared to those in healthy controls^27,28^. Interestingly, a higher *BDNF* promoter methylation status has been associated with suicidal ideation and suicidal attempt history in depressive patients^29^.

Regarding the influence of external factors on gene regulation through DNA methylation, there is evidence that DNA methylation of the *BDNF* gene is modified by early life negative stressors^30,31^. Moreover, differences are observed in specific brain areas and in a sex-specific manner^32^. Other factors that have been observed to influence *BDNF* methylation are tobacco consumption^33^, age^34^, and pharmacological treatment, with higher methylation levels of *BDNF* promoter I in patients under treatment with antidepressants^35^.

To our knowledge, there are no previous studies addressing the involvement of BDNF regulation on cognitive performance in MDD. We hypothesize that specific methylation of promoter regions of the BDNF will be associated to poorer performance in neurocognitive tasks. Our goal was to study the association between BDNF genetic and epigenetic variation with neurocognitive performance and to explore the influence of each specific CpG site within the BDNF gene promoters on cognition, taking into account potential modulating external factors and confounders.

## Materials and methods

### Study sample

The sample consisted of 64 MDD patients (72% females, mean age 57.1 ± 1.3 years) diagnosed according to DSM-IV-TR criteria (American Psychiatric Association, 2000) and 71 healthy controls (HCs) (63% females, mean age 54.2 ± 1.3 years. MDD patients were recruited from the Psychiatry Department at Bellvitge University Hospital (Hospitalet de Llobregat, Barcelona) while HCs were recruited from the same geographic region through advertisements. All participants were unrelated Caucasians of Iberian Peninsula ancestry.

The exclusion criteria were as follows: age less than 18 years, non-Caucasian ethnicity, a diagnosis of other psychiatric disorders including substance abuse or dependence (except nicotine), neurological disorders including dementia, mental retardation, severe medical conditions, electroconvulsive therapy in the previous year, pregnancy or puerperium, and corticosteroid treatment in the previous 3 months.

The sample partially overlaps that used in previous studies^36,37^, which explored different hypotheses. The current participants agreed to provide blood samples for DNA extraction, genotyping, and methylation studies. The Clinical Research Ethics Committee (CEIC) of the Bellvitge University Hospital approved the research protocol, all participants provided written informed consent after having received a full explanation of the study.

### Clinical assessment

All patients met DSM-IV-TR criteria for MDD and were interviewed by experienced psychiatrists using the Mini-International Neuropsychiatric Interview (MINI)^38^. HCs were required to have no history of present or past psychiatric disorders and to show a score lower than 7 in the 28-item Spanish adaptation of the Goldberg General Health Questionnaire (GHQ-28). A semistructured interview was administered to all participants to asses sociodemographic and clinical variables, substance use and treatments. Antidepressant treatment was recorded using the World Health Organization Anatomical Therapeutic Chemical Classification System^39^, which measures treatment as the defined daily dose (DDD), that is, the assumed average maintenance dose per day for a drug used for its main indication in adults.

The 17-item Hamilton Depression Rating Scale (HDRS) was used to assess depression severity^40^. Treatment resistance in the MDD group was assessed using the staging model as proposed by Thase and Rush^41^, which defines 5 levels of resistance according to the number and classes of antidepressants that have failed to produce a response. The state-trait anxiety inventory (STAI) was used to evaluate anxiety both in patients and HCs^42^. The Childhood Trauma Questionnaire (CTQ), which examines the exposure to five types of trauma in childhood and has been previously validated in healthy and clinical populations, was administered to all participants^43^. Three types of childhood maltreatment are focused on abuse (sexual, physical, and emotional) while two types of maltreatment are centered on neglect (physical and emotional). Each type of maltreatment is measured using five items and each item is rated on a five-point scale.

### Neuropsychological assessment

The Spanish version of the Mini-mental State Examination (MMSE) was used as a screening for dementia. The following neuropsychological tests were administered to all participants to assess different cognitive domains: (1) verbal learning and memory: Hopkins Verbal Learning Test Revised^TM^ (HVLT-R); (2) visual learning and memory: Brief Visuospatial Memory Test Revised^TM^ (BVMT-R) and the Rey Complex Figure Test (RCFT), which includes copy, immediate recall, and delayed recall subscores; (3) working memory: Corsi Block-Tapping Test (CBTT) and Letter-Number Span (LNS); (4) processing speed: Trail Making Test Part A (TMT-A), Brief Assessment of Cognition in Schizophrenia – Symbol Coding (BACS-SC), and category fluency (animal naming); (5) attention/vigilance: Continuous Performance Test – Identical Pairs (CPT-IP);(6) selective attention/interference: Stroop test (direct subscores for words, W; colors, C; words-colors, WC; interference); (7) reasoning and problem solving: Neuropsychological Assessment Battery® Mazes (NAB-Mazes); (8) executive control: Trail Making Test Part B (TMT-B). In all tests, higher scores reflected better cognitive performance with the exceptions of TMT-A and TMT-B, where the outcome measure is the number of seconds needed to perform the task; thus, higher scores reflected poorer cognitive performance.

### SNP selection and genotyping

Blood samples were obtained close to the neuropsychological assessment. The Tagger tool of Haploview v.4.2^44^ was used to select tagSNPs in linkage disequilibrium (LD) (*r*^2^ > 0.7) with the remaining SNPs at minor allele frequency (MAF) > 10% from the HapMap phase III European samples. Functional SNP rs6265 and SNPs most commonly associated with mental disorders or cognitive dysfunction from the literature (rs2030324, rs12273363, rs908867, and rs1491850) were prioritized as tagSNPs^45–48^. In addition, rs11030094, rs11602246, and rs4923463, which were absent from the HapMap data, were included following Hennings, Honea, and Neves^49–51^. In total, 11 SNPs were selected (Fig. S1).

Genotyping of the selected SNPs was performed using the MassARRAYiPLEX platform (Agena Bioscience, formerly Sequenom, Inc.; San Diego, California). We used mean methylation levels from the performed technical replicas and discarded outlying values (standard deviation >10%) as a quality control method. The genotyping assays were performed at the genotyping facilities of CeGen in the Santiago de Compostela Node (‘Centro Nacional de Genotipado’).

### Selection of genomic regions and quantitative DNA methylation analysis

We selected promoters I and IV for our methylation study analyses since these are promoters with more consistent associations with psychiatric disorders^27^. We used two assays to cover promoter I, with Assay 1 covering the region chr11: 27744025-27744278 and Assay 2 covering the region chr11: 27744414-27744653, and one assay for promoter IV, covering chr11: 27722305-27722675 (UCSC h19 assembly). The CpG sites analyzed in each region can be found in Table S1 of the Supplementary Material.

DNA purification, bisulfite treatment, and quantitative DNA methylation analysis using the MassArray platform of SEQUENOM were performed as described^52^. Primers were designed using MethPrimer (http://www.urogene.org/ methprimer/). Primers were tagged to obtain an appropriate product for in vitro transcription, prevent abortive cycling and balance the PCR primer length (forward primer tag: cagtaatacgactcactatagggagaaggct, reverse primer tag: aggaagagag). The sequences of the primers used for amplification are shown in Table S1 of the Supplementary Material.

### Statistical analyses

We processed data using SPSS 19.0 (SPSS, IBM, USA) to perform descriptive and univariate analyses comparing the MDD and HCs groups using the *χ*^2^ test for dichotomic categorical variables and Student’s *t*-test for continuous variables. We previously analyzed the distribution of variables, and those following a skewed distribution were log transformed (ln) to approximate normality, which was the case for the HDRS and CTQ scores and two cognitive tests (TMT-A and TMT-B).

Association analysis at the individual SNP level was performed as implemented in PLINK 1.9^53^ to conduct multivariate analysis based on linear regression. Statistical significance was assessed by a permutation procedure to estimate the significance of the best result (10,000 permutations). Three different genetic models (dominant, recessive, and additive) were considered. These analyses represent an effective number of tests of 2.2^54^; therefore, the results were considered experiment-wise significant if *p* value < 0.023 after the permutation procedure. The analyses included sex, age, years of education, MDD diagnosis, tobacco consumption, HDRS, STAI trait subscore and CTQ score as covariates.

An analysis of plausible mediation through methylation variables in the relationship between SNPs and neurocognitive variables was performed with the PROCESS macro of SPSS version 21.0, developed by Hayes^55^. This method uses bias-corrected bootstrap confidence intervals. The results were considered significant when the bias-corrected 95% confidence intervals did not contain zero.

Partial correlation analyses adjusted by age, gender, and years of education were used to explore the relationship between methylation measures and cognitive performance. We conducted a stratified analysis by diagnosis (HCs vs. MDD). In the MDD group, we also included relevant clinical variables (HDRS, CTQ score, STAI trait sub-score and antidepressant treatment).

Multiple linear regression analyses were carried out in all participants to explore the association between methylation variables and neuropsychological performance, the latter being considered as dependent variables. Separate multiple regression analyses were performed for each methylation measure, each of which was considered the main independent variable. We controlled for covariates and potential confounders including sex, age, years of education, tobacco consumption, diagnostic group, HDRS, STAI-trait sub-score and CTQ score. Antidepressant treatment was not included as a covariate since this variable presents a high collinearity with MDD diagnosis. Finally, we explored the association between neuropsychological results and the interactions between methylation status with sex, CTQ score and MDD diagnosis. Standardized beta coefficients will be reported. Standardized beta coefficients are calculated by subtracting the mean from the variable and dividing by its standard deviation. This results in standardized variables having a mean of zero and a standard deviation of 1. The standardization allows the comparison of all independent variables in the equation. Standardized beta coefficients reflect how many standard deviations a dependent variable changes, per standard deviation increase in the predictor (independent) variable. As the standardized beta coefficient reflects the strength of the association between an independent variable (e.g. methylation of the promoter IV of the BDNF gene) and the dependent variable (e.g. visual memory task), a positive beta coefficient would indicate a better cognitive performance in those individuals with a greater methylation whereas a negative beta coefficient would indicate a poorer cognitive performance in those individuals with a greater methylation.

The main analyses testing the hypotheses related to the role of methylation of promoter I and IV of the BDNF gene (mean methylation values) on each cognitive test (17 different cognitive measures) were adjusted for multiple comparisons with the Benjamini-Hochberg procedure, that allows controlling for the false discovery rate (FDR)^56^. The results will be adjusted for different FDR (20%, 10%, and 5%) taking into account 34 comparisons (17 cognitive variables × 2 promoters). We further performed statistical analyses under an exploratory approach when studying association between specific *BDNF* methylation CpGs and cognitive performance. For these analyses, the statistical significance level was set at *p* < 0.05 (bilateral), without an adjustment for multiple comparisons, as correction for multiple testing is not strictly necessary in those analyses that are exploratory in nature^57^.

## Results

### Univariate analysis

Demographic and clinical variables are shown in Table 1. As expected, MDD patients showed higher HDRS scores than HCs. MDD patients also showed significantly higher scores in the CTQ and STAI trait subscore. In the cognitive functioning analysis, MDD patients showed poorer cognitive performance than HCs on all cognitive domains (Table 2). Mean methylation values at each CpG site are shown in Table S2 of the Supplementary Material. Methylation levels were higher in HCs than in MDD with significant differences in CpG 1 and 7.8.9 of promoter I assay 1, CpG 14 of promoter I assay 2 and CpG 11, 13 and 15.16.17 of promoter IV.

**Table 1:** Demographic and clinical data of the study sample

	HCs (*n* = 70)	MDD (*n* = 64)	*p* value
Age (years)	54.5 (10.5)	57.1 (10.6)	0.171
Female gender, *n* (%)	44 (62.9%)	46 (71.9%)	0.267
Education (years)	11.6 (3.3)	9.7 (4.2)	0.004
Tobacco consumption (cigarettes/day)	2 (5.3)	4.1 (8.6)	0.089
HDRS	0.7 (1.1)	12.3 (9.1)	<0.001
Antidepressant treatment (DDD)	0 (0)	2.3 (1.3)	<0.001
Treatment resistance (Thase)^a^	–	–	–
Stage 0		20 (31.3%)	
Stage I		13 (20.3%)	
Stage II		10 (15.6%)	
Stage III		12 (18.8%)	
Stage IV		9 (14.1%)	
Stage V		0 (0%)	
Childhood trauma questionnaire^b^	34.3 (9.7)	38.5 (14.1)	0.043
STAI trait score	15.3 (8.6)	32.3 (13.7)	<0.001

All variables presented in mean (SD), or *n* (%). Missing data: CTQ (1.5%), STAI (1%)

*HCs* Healthy controls, *MDD* major depressive disorder, *HDRS* Hamilton Depression Rating Scale, *DDD* Defined Daily Doses

^a^Criteria for treatment resistance stages were: non-resistant or any medication trials, to date, judged to be adequate (Stage 0); failure of at least one adequate trial of one major class of antidepressant (Stage I); failure of at least two adequate trials of at least two distinctly different classes of antidepressants (Stage II); Stage II resistance plus failure of an adequate trial of a tricyclic antidepressant or a first augmentation strategy (lithium or thyroid hormone) (Stage III); Stage III resistance plus failure of an adequate trial of an MAOI or a second augmentation strategy (Stage IV); Stage IV resistance plus failure of an adequate course of bilateral electroconvulsive therapy (Stage V)

**Table 2:** Neuropsychological performance in major depressive disorder patients and healthy controls

	HCs (*n* = 70)	MDD (*n* = 64)	*p* value
Cognitive domains			
Verbal learning and memory			
HVLT-R	23.86 (4.92)	20.37 (5.17)	<0.001
Visual learning and memory			
BVMT-R	22.66 (7.52)	14.72 (9.08)	<0.001
RCFT – copy	30.84 (5.34)	27.70 (7.42)	0.006
RCFT – immediate recall	17.45 (6.36)	12.66 (6.57)	<0.001
RCFT – delayed recall	17.76 (6.31)	12.13 (6.78)	<0.001
Working memory			
CBTT (non-verbal)	14.45 (3.74)	11.59 (3.76)	<0.001
LNS (verbal)	13.72 (3.41)	10.58 (3.49)	<0.001
Processing speed			
TMT-A^a^ (seconds)	45.71 (24.00)	65.76 (38.41)	<0.001
BACS-SC	47.73 (13.8)	33.11 (15.89)	<0.001
Category fluency	23.74 (6.53)	20.05 (6.26)	0.001
Stroop Direct W	100.90 (15.81)	92.83 (22.67)	0.021
Stroop Direct C	70.93 (12.17)	61.17 (14.68)	<0.001
Attention/vigilance			
CPT-IP	2.52 (0.87)	1.99 (0.88)	0.002
Executive function			
TMT-B^a^ (seconds)	82.24 (48.83)	149.19 (124.23)	<0.001
NAB-Mazes	14.80 (6.86)	8.95 (7.19)	<0.001
Stroop Direct WC	45.06 (12.76)	36.92 (11.04)	<0.001
Stroop Direct Interference	3.62 (9.59)	0.38 (6.87)	0.026

All variables presented in mean (SD), or *n* (%). Missing data differed for cognitive tests: HVLT-R (0.7%), BVMT-R (2.2%), LNS (4.4%), TMT A (1.5%), BACS-SC (1.5%), Category fluency (0.7%), Stroop Direct W and C (0.7), CPT-IP (13.4%), TMT-B (4.5%), NAB-Mazes (2.2%), Stroop Direct WC and Interference (1.5%)

*HCs* healthy controls, *MDD* major depressive disorder, *HVLT-R* Hopkins Verbal Learning Test-Revised, *BVMT-R* Brief Visuospatial Memory Test-Revised, *RCFT* Rey Complex Figure Test, *CBTT* Corsi Block-Tapping Test, *LNS* Letter Number Span, *TMT-A* Trail Making Test part A, *BACS-SC* Brief Assessment of Cognition in Schizophrenia-Symbol Coding, *W* words, *C* colors, *CPT-IP* Continuous Performance Test-Identical Pairs, *TMT-B* Trail Making Test part B, *NAB-Mazes* Neuropsychological Assessment Battery-Mazes, *WC* words-colors

^a^TMT-A and TMT-B raw scores are shown. *P* values calculated upon natural log-transformed variables

### Association analyses of individual SNPs

All genotyped polymorphisms were in Hardy-Weinberg equilibrium and had call rates higher than 90%. After correction for multiple testing using a 10,000 permutation procedure, two SNPs showed an association with cognitive performance under a dominant model: rs908867-T (standardized *β*: 0.228; *P* value: 0.013) was associated with the BVMTR test outcome, and rs925946-T (standardized *β*: 0.165; *P* value: 0.008) was associated with the BACS-SC result. No other associations between individual SNPs and cognitive performance were detected. We also tested for possible interactions between significant SNPs with sex, CTQ and MDD diagnosis. One significant result was found between the interaction of rs925946-T and MDD diagnosis with the BACS-SC result (MDD diagnosis [*β*: −0.25; *p* value: 0.005], rs925946 [*β*: 0.081; *p* value: 0.226], interaction MDD x rs925946-T [*β*: 0.171; *p* value: 0.035]). We also performed post hoc analyses of the association between individual SNPs and treatment resistance in the MDD group but found no significant association.

### Mediation analysis

A significant mediation was found in the association between rs908867 and the BVMTR test results through differential methylation at the CpG 7.8.9 site of promoter I. The bias-corrected bootstrap 95% CI indicated that the indirect effect through methylation differences was significant using a dominant model (Effect: 0.84 (SE 0.49), 95% CI, [0.15, 2.24]). No other significant mediations were found.

### Partial correlation analyses

We analyzed the correlation between methylation variables and neuropsychological performance. The correlation heat map of these partial correlation analyses stratified by diagnosis is included in Figs. S2 and S3 of the Supplementary Material. Antidepressant treatment was not correlated with methylation measures.

### Multiple linear regression analyses

The results of multiple linear regression analyses of mean methylation levels and neuropsychological performance in all participants are shown in Table 3.

**Table 3:** Results of multiple linear regression analyses of mean methylation levels and neuropsychological performance in all participants

	Promoter I			Promoter IV
	Mean	Assay 1	Assay 2	Mean
	*β*	*β*	*β*	*β*
Verbal learning and memory				
HVLT-R	0.047	0.100	–0.030	–0.013
Visual learning and memory				
BVMT-R	0.105	–0.042	–0.144	–0.152^*^
RCFT- copy	0.039	0.102	–0.047	–0.111
RCFT - immediate recall	–0.060	<0.001	–0.108	–0.240^**^
RCFT - delayed recall	–0.031	–0.009	–0.046	–0.261^***^
Working memory				
CBTT (non-verbal)	0.089	0.083	0.065	–0.014
LNS (verbal)	–0.193^**^	–0.129	–0.207^**^	–0.177^*^
Processing speed				
TMT - A	–0.011	–0.045	0.031	0.106
BACS SC	0.002	–0.022	0.029	–0.048
Fluency	–0.014	–0.029	0.009	–0.175^*^
Stroop Direct W	0.049	0.080	–0.004	0.068
Stroop Direct C	0.054	0.059	0.029	–0.029
Attention/vigilance				
CPT-IP	0.032	0.069	−0.019	−0.046
Executive function				
TMT- B	–0.069	–0.053	–0.066	0.061
NAB Mazes	0.006	0.026	–0.019	–0.124
Stroop Direct WC	0.105	0.087	0.091	<0.001
Stroop Direct Interference	0.107	0.062	0.125	–0.010

Statistically significant results are highlighted as bold (^*^p < 0.05; ^**^p < 0.01;^***^p < 0.001).

Linear regressions were adjusted by sex, age, years of education, MDD diagnosis, tobacco consumption, HDRS, STAI trait score and CTQ score.

*β* Standardized beta coefficient, *HVLT-R* Hopkins Verbal Learning Test-Revised, *BVMT-R* Brief Visuospatial Memory Test-Revised, *RCFT* Rey Complex Figure Test, *CBTT* Corsi Block-Tapping Test, *LNS* Letter Number Span, *TMT-A* Trail Making Test part A, *BACS-SC* Brief Assessment of Cognition in Schizophrenia-Symbol Coding, *W* words, *C* colors, *CPT-IP* Continuous Performance Test-Identical Pairs, *TMT-B* Trail Making Test part B, *NAB*-Mazes Neuropsychological Assessment Battery-Mazes, *WC* words-colors, *MDD* major depressive disorder, *HDRS* Hamilton Depression Rating Scale, *STAI* State-Trait Anxiety Inventory, *CTQ* Childhood Trauma Questionnaire

#### Promoter I

A higher promoter I mean methylation level was associated with poorer performance in the LNS test, a result which remained significant with a FDR of 20% but did not reach significance at a FDR of 10%. This association was also observed when analyzing methylation levels in the region covered at Assay 2, while it did not appear in Assay 1. The results of the multiple linear regression analyses of each CpG site in promoter I can be found in Tables S3 and S4 of the Supplementary Material. In Assay 1, CpG site 7.8.9 was associated with poor results in most cognitive domains (visual learning and memory, working memory, processing speed, attention/vigilance, and executive functioning). An inverse relationship was observed in CpG site 10, where higher methylation predicted better results in visual learning and memory, processing speed, attention/vigilance, and executive functioning tests. In Assay 2, poorer cognitive functioning in the LNS test was related to higher mean methylation levels at CpG sites 3.4.5.6 and 9. Another significant association was observed between higher methylation values at the CpG 11 site and poor performance in the BVMT-R test.

#### Promoter IV

Mean methylation levels at this promoter location were significantly associated, at a FDR of 20%, to poorer performance in BVMT-R, RCFT immediate and delayed recall, LNS and fluency tests. However, at a 5% FDR only RCFT immediate and delayed recall were significant. Results of multiple linear regression analyses of each CpG site in promoter IV can be found in Table S5 of the Supplementary Material. CpG sites 5, 9, and 10 presented an association with poorer cognition in several cognitive domains (verbal and visual learning and memory, working memory, processing speed and executive functioning). Additionally, CpG site 13 showed an especially significant association with worse performance in visual learning and memory tasks (RCFT copy, immediate, and delayed recall).

### Interaction analysis

The results of the analyses of significant interactions between methylation variables and sex, CTQ score and MDD diagnosis are shown in Table S6 of the Supplementary Material. Regarding sex, the most consistent interaction was found between CpG site 22.23 of promoter IV, in which higher methylation values were associated with better cognitive performance in women, whereas an inverse association was observed in men. Regarding interactions between methylation variables and CTQ score, most significant interactions suggested that a combination of higher methylation and a greater history of childhood trauma were associated with poorer cognitive functioning in attention, executive function and visual and verbal memory. Finally, when exploring the interaction with MDD diagnosis, we found that in both promoter I and IV and in different cognitive domains, significant associations between higher methylation and the presence of MDD diagnosis could be found, where higher methylation values were associated with poorer cognition in MDD patients, while the inverse association was found in HCs.

## Discussion

We have studied the contribution of *BDNF* genetic and epigenetic variations to cognitive functioning in MDD patients and HCs. Our main findings include the association between two SNPs as well as promoter methylation levels with several cognitive tasks and statistically significant interactions with sex, CTQ scores and MDD diagnosis. In addition, differential methylation at the CpG 7.8.9 site of BDNF promoter I mediated the association between the individual SNP rs908867 and visual memory.

In the present study exploring the relationship between 11 *BDNF* SNPs and cognitive functioning, two noncoding SNPs (rs908867 and rs925946) were associated with poorer performance on cognitive tasks dealing with visual learning, memory and processing speed. Although most research has focused on the role of the functional SNP rs6265 in cognitive performance, there is evidence that SNPs located in noncoding regions can also influence gene regulation^58^. In this line, a well-characterized noncoding SNP within *BDNF* is rs12273363, which has been shown to regulate *BDNF* transcription by altering promoter IV activity^59^. Although this particular SNP was not associated in our sample, our findings related to associations with two noncoding SNPs (rs908867 and rs925946) lend support to the hypothesis that noncoding SNPs may also influence *BNDF* activity. Several associations have been found relating *BDNF* SNP rs908867 with a variety of clinical and neuroimaging phenotypes, including suicidality^60^, treatment response in MDD^48^ and hippocampal and cerebral atrophy^49^. Interestingly, in the study by Januar and colleagues^27^, only major homozygous patients showed an association between *BDNF* promoter methylation and depression, and for CpG site 7.8.9, a trend association was observed with depression, with rs908867 significantly modifying this relationship. Our results support the evidence of a relationship between rs908867 and clinical phenotypes, including cognitive dysfunction, an effect that seems to be mediated by methylation at the CpG 7.8.9 site of BDNF promoter I. One study including healthy individuals found an association between rs925946 and impairments in long-term visual memory tasks^61^. Future studies focusing on the influence of these SNPs on *BDNF* transcription regulation are needed to describe the pathway through which they could be involved in cognitive function.

Exploring the relationship between *BDNF* gene regulation through methylation and neurocognitive performance showed that, while higher methylation levels of most regions were associated with poorer cognitive performance in the whole sample and across all cognitive domains, the effect of methylation in certain areas, such as CpG site 10 in promoter I, was associated with better cognitive function. These results suggest that hypermethylation at CpG site 10 could have a protective neurocognitive effect. Another interesting association was found in the promoter IV region, in which CpG site 13 showed a strong and specific association with impaired visual learning and memory, while CpG sites 3, 9, and 10 seemed to be associated with deficits in multiple areas. The mechanism through which differential methylation of CpG site 13 can have a specific impact on visual learning and memory, while other sites have a broader cognitive effect, should be the focus of future studies. Taken as a whole, our findings suggest an effect of *BDNF* gene promoter methylation and neurocognitive performance. Our results are in accordance with previous data from epigenome-wide meta-analyses that reported an association between DNA methylation in different genomic areas and cognitive performance^62^.

To date, many studies have focused on the biological mechanisms through which *BDNF* expression can influence cognitive performance. Previous studies based on animal models examined the influence of *BDNF* variations on different cognitive processes^63–66^. Some studies have shown an association between *BDNF* gene methylation and an increased risk of developing neurodegenerative disorders^67,68^, but the results were not replicated in others^69^.

The cognitive tasks most frequently associated with methylation variables in our results are related to memory processes (visual, verbal, and working memory), which are thought to be mediated by the hippocampus and prefrontal cortex. The mechanisms that regulate hippocampal function in memory have been widely studied. Among other factors, *BDNF* is a key protein involved in hippocampal neurogenesis^70,71^. *BDNF* function is subject to epigenetic control, which is sensitive to external modulating factors, including stress and childhood trauma^72^. In this regard, animal studies have shown that adolescent trauma is associated with elevated corticosterone levels and lower levels of *BDNF* in the dorsal hippocampus^73^. DNA methylation represents an epigenetic mechanism that contributes to the regulation of *BDNF* transcription in the CNS and is therefore involved in memory formation^66^. Research focusing on the influence of *BDNF* methylation and structural brain variations in MDD patients has described associations with cortical thickness, particularly in the prefrontal and occipital areas^74^, hippocampal atrophy^49^ and white matter integrity in the anterior corona radiata^75^.

Our results also show that the association between *BDNF* methylation and cognition can be moderated by sex, childhood trauma or MDD diagnosis. In reference to sex differences, previous human studies^76^ have reported higher levels of DNA methylation of promoter IV of *BDNF* in females. In line with these findings, animal studies exploring whether maltreatment or nurturing care is associated with differential methylation of BDNF within the medial prefrontal cortex of adult rats have demonstrated that methylation of DNA associated with exon IV is increased in female maltreated rats^31^. Interestingly, in our study, we found significant interactions between methylation values and female sex with several cognitive tasks dealing with executive function and processing speed, which are known to involve the prefrontal cortex.

No previous studies have explored the moderation effect of childhood trauma on the relationship between *BDNF* methylation and cognitive functioning. In a recent twin study, DNA methylation in several stress-related genes, including *BDNF*, mediated the association between childhood trauma and depressive symptoms at adulthood^77^. Animal studies have also demonstrated that early life adversity produces persistent changes in DNA methylation of *BDNF* that cause altered *BDNF* gene expression in the adult prefrontal cortex^30^. Studies in humans have shown an effect of childhood abuse on the epigenetic regulation of hippocampal glucocorticoid receptor expression, which could lead to cognitive dysfunction^78^.

Although the aim of our study was not to analyze differences in *BDNF* methylation between diagnostic groups, we observed significantly higher methylation levels at specific promoter regions of the *BDNF* gene in HCs compared to MDD patients, results which contrast with previous findings showing greater methylation in patients^26,27^. MDD diagnosis moderated the association between methylation of *BDNF* and cognitive functioning in memory and executive function, as MDD patients with increased methylation showed a poorer cognitive profile. As there are no previous studies exploring this issue in relation to cognition, it is difficult to draw definitive conclusions. Our findings suggest that methylation of *BDNF* may contribute to the persistent cognitive deficits observed in MDD patients.

To our knowledge, the present study is the first to explore the effect of *BDNF* regulation through DNA methylation on cognitive performance while adjusting for modulating factors in MDD patients and HCs. Our results suggest a relationship between methylation levels at specific promoter locations and cognitive function. Therefore, this work suggests that future research on *BDNF* regulation might help identify specific groups of MDD patients who might benefit the most from intensive antidepressant treatment targeting cognitive symptoms or neurocognitive rehabilitation.

There are some limitations of our study that merit discussion. First, we need to underscore that the association analysis between specific CpG sites and cognitive performance was exploratory in nature. Thus, hypotheses generated by these results need to be tested in further confirmatory studies. Second, the small sample size of our study might have reduced the statistical power to detect small effect sizes. Third, clinical variables such as childhood trauma and anxiety questionnaires were retrospectively self-reported and could be influenced by recall bias and depressive state. Fourth, patients were recruited in a naturalistic setting, and therefore relevant differences in clinical variables such as educational level existed between groups. For this reason, we adjusted all analyses by the factors that showed significant differences between groups and that could influence gene methylation according to previous literature. For example, a recent meta-analysis reported an association between methylation at three specific genomic sites and depressive symptomatology; therefore, all analyses were adjusted by HDRS results^79^. Moreover, MDD patients were receiving antidepressant treatment, which has been suggested to influence DNA methylation at the *BDNF* gene^35^. However, no significant association between antidepressant treatment dose (DDD) and DNA methylation at the BDNF gene was identified in our sample. Fifth, patients recruited from a tertiary source may differ from community-based cases, which could limit the generalization of results. Finally, we assessed the levels of methylation at the *BDNF* gene in peripheral blood cells. Although it might be argued that methylation studied in peripheral blood cells could be different than methylation in the brain, recent studies suggest that peripheral BDNF methylation closely reflects that of brain tissues^80^.

In summary, our study provides further insight into the relationship between *BDNF* and cognitive performance, suggesting that methylation of the *BDNF* gene influences hippocampus- and prefrontal cortex-mediated cognitive tasks and that female sex, childhood trauma and MDD are moderators of these associations.

## Supplementary information

Figure S1

Table S1

Table S2

Figure S2

Figure S3

Table S3

Table S4

Table S5

Table S6
