# Supplementary figures and images for: BDNF genetic variants and methylation: effects on cognition in major depressive disorder
Source: Transl Psychiatry. 2019 Oct 21;9:265. doi: 10.1038/s41398-019-0601-8 (PMC6803763; doi:10.1038/s41398-019-0601-8)

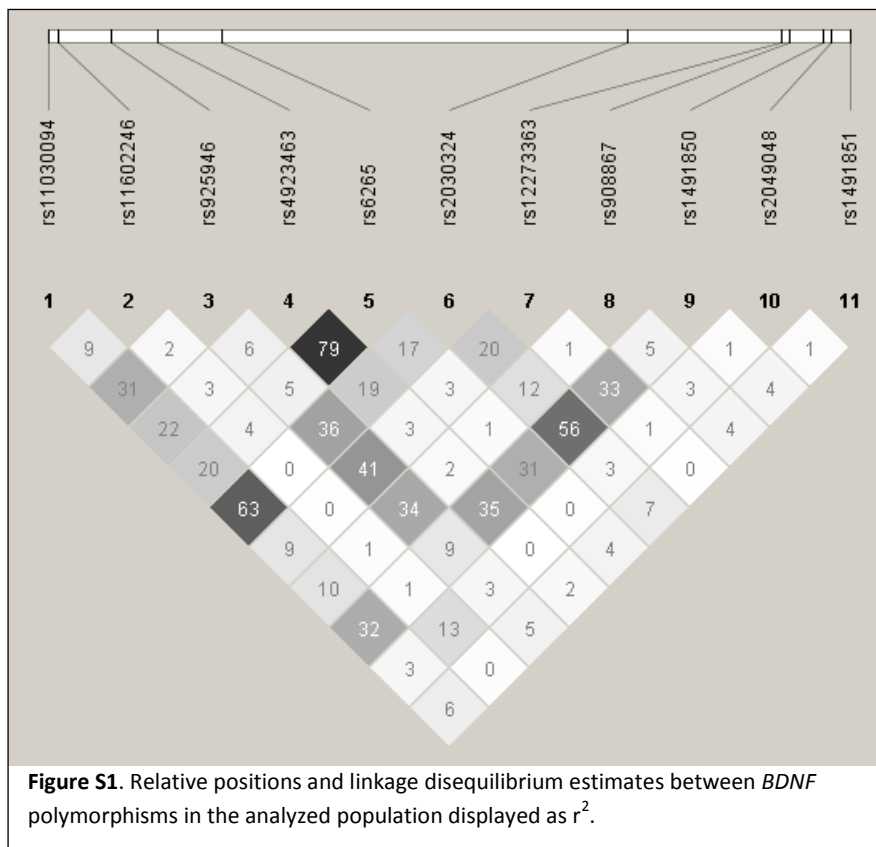

Supplement: Supplementary file 1 — Figure S1 [file 41398_2019_601_MOESM1_ESM.pdf]
