# Supplementary material for: BDNF genetic variants and methylation: effects on cognition in major depressive disorder
Source: Transl Psychiatry. 2019 Oct 21;9:265. doi: 10.1038/s41398-019-0601-8 (PMC6803763; doi:10.1038/s41398-019-0601-8)
Supplement: Supplementary file 2 — Table S1 [file 41398_2019_601_MOESM2_ESM.pdf]

Table S1. Genomic sites of methylation interrogated in the current study.

Amplicon	Genomic location	Primers	Analytic Unit	Unit Location
BDNF promoter I				
Assay1	27744025-27744278	forward: TAGGGTTTTTTGGGAGAGTTTTTT	CpG-1	25
		reverse: TTCCCAAATATAAATTAACAACCCC	CpG-6	55
			CpG-7.8.9	62, 68, 70
			CpG-10	86
			CpG-11.12	98, 110
			CpG-15	139
			CpG-26	228
Assay2	27744414-27744653	forward: TTTTTTGGAGTGTTTAATAGAGGGG	CpG-1	30
		reverse: AATCTACATTCATCCCTAATTCTTC	CpG-2	42
			CpG-3.4.5.6	50,53,60,62
			CpG-9	92
			CpG-11	124
			CpG-12	144
			CpG-14	198
BDNF promoter IV	27722305-27722675	forward: TGAATTTTTATTAGGTAAATTTTAGAGAGG	CpG-3.4	41,46
		reverse: AAAAATATTCCAACCCCAACCT	CpG-5	48
			CpG-6.7.8	66, 76, 84
			CpG-9	94
			CpG-10	105
			CpG-11	109
			CpG-13	122
			CpG-15.16.17	191, 198, 219
			CpG-20	249
			CpG-21	251
			CpG-22.23	268, 313

As per hg19 nomenclature.
