# Supplementary material for: BDNF genetic variants and methylation: effects on cognition in major depressive disorder
Source: Transl Psychiatry. 2019 Oct 21;9:265. doi: 10.1038/s41398-019-0601-8 (PMC6803763; doi:10.1038/s41398-019-0601-8)
Supplement: Supplementary file 3 — Table S2 [file 41398_2019_601_MOESM3_ESM.pdf]

Table S2.
Methylation values at specific CpG sites by diagnostic group.

		HC (n = 70)	MDD (n = 64)	p value
Promoter I				
Assay 1				
	CpG_1	3.69 (1.29)	3.13 (1.19)	0.01
	CpG_6	2.91 (0.97)	2.81 (0.87)	0.53
	CpG_7_8_9	2.00 (0.87)	1.69 (0.64)	0.02
	CpG_10	4.53 (2.75)	4.92 (2.69)	0.40
	CpG_11_12	3.64 (1.12)	3.33 (1.18)	0.12
	CpG_15	3.97 (2.22)	3.92 (2.18)	0.90
	CpG_26	3.76 (1.50)	3.70 (1.62)	0.84
Assay 2				
	CpG_1	0.97 (1.18)	0.77 (1.14)	0.31
	CpG_2	2.36 (0.83)	2.31 (0.66)	0.73
	CpG_3_4_5_6	8.63 (2.27)	8.42 (2.41)	0.61
	CpG_9	1.56 (0.85)	1.41 (0.83)	0.30
	CpG_11	4.01 (0.94)	4.03 (0.85)	0.91
	CpG_12	2.09 (1.64)	1.77 (1.24)	0.21
	CpG_14	12.51 (2.71)	10.03 (2.58)	<0.01
Promoter IV				
	CpG_3_4	3.37 (0.90)	3.27 (0.90)	0.50
	CpG_5	3.21 (1.19)	3.27 (1.10)	0.80
	CpG_6_7_8	5.37 (2.03)	4.95 (1.86)	0.22
	CpG_9	2.11 (1.17)	2.30 (1.49)	0.43
	CpG_10	1.64 (0.96)	1.69 (1.05)	0.80
	CpG_11	8.74 (1.16)	8.31 (1.15)	0.03
	CpG_13	5.61 (1.60)	5.02 (1.78)	0.04
	CpG_15_16_17	6.83 (1.04)	6.09 (1.00)	<0.01
	CpG_20	1.47 (1.20)	1.53 (1.05)	0.76
	CpG_21	4.90 (1.05)	5.03 (0.91)	0.44
	CpG_22_23	1.76 (0.69)	1.61 (0.81)	0.26

Abbreviations: HC, Healthy controls; MDD, major depressive disorder
All variables presented in mean (SD).
