# Supplementary material for: BDNF genetic variants and methylation: effects on cognition in major depressive disorder
Source: Transl Psychiatry. 2019 Oct 21;9:265. doi: 10.1038/s41398-019-0601-8 (PMC6803763; doi:10.1038/s41398-019-0601-8)
Supplement: Supplementary file 4 — Figure S2 [file 41398_2019_601_MOESM4_ESM.pdf]

Figure S2

Heatmap of partial correlation analyses between methylation variables and neuropsychological measures in major depressive disorder patients.

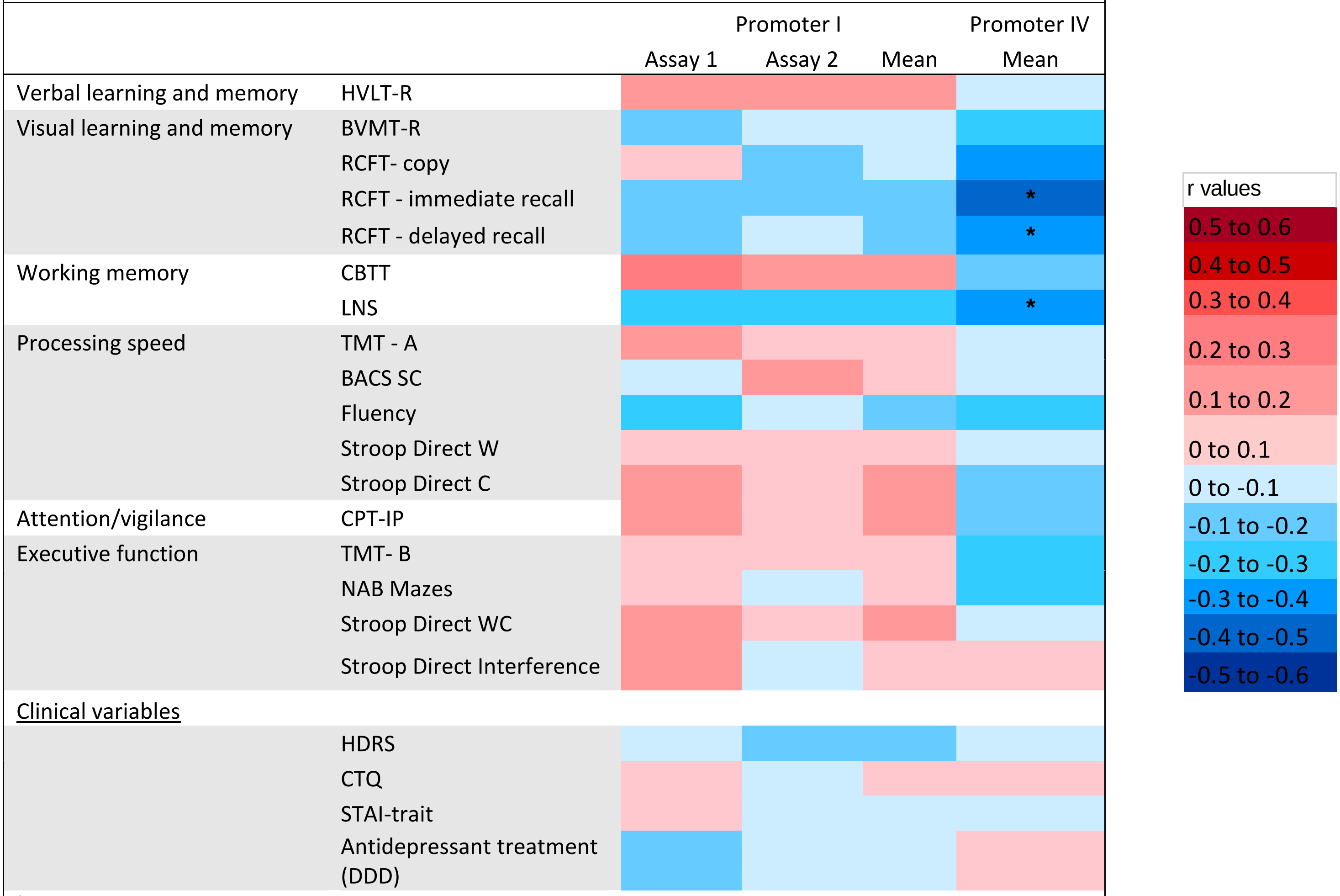

*Statistically significant results ($p<0.05$)

TMT-A and TMT-A r values are inverted (since higher results in these tests imply a worse cognitive function).

Abbreviations: HVLT-R, Hopkins Verbal Learning Test-Revised; BVMT-R, Brief Visuospatial Memory Test-Revised; RCFT, Rey Complex Figure Test; LNS, Letter Number Span; TMT-A, Trail Making Test part A; BACS-SC, Brief Assessment of Cognition in Schizophrenia-Symbol Coding; W, words; C, colors; CPT-IP, Continuous Performance Test-Identical Pairs; TMT-B, Trail Making Test part B; NAB-Mazes, Neuropsychological Assessment Battery-Mazes; WC, words-colors; HDRS, Hamilton Depression Rating Scale; STAI, State-Trait Anxiety Inventory; CTQ, Childhood Trauma Questionnaire; DDD, Defined Daily Dose.
