# Supplementary material for: BDNF genetic variants and methylation: effects on cognition in major depressive disorder
Source: Transl Psychiatry. 2019 Oct 21;9:265. doi: 10.1038/s41398-019-0601-8 (PMC6803763; doi:10.1038/s41398-019-0601-8)
Supplement: Supplementary file 5 — Figure S3 [file 41398_2019_601_MOESM5_ESM.pdf]

Figure S3

Heatmap of partial correlation analyses between methylation variables and neuropsychological measures in healthy controls.

		Promoter I			Promoter IV
		Assay 1	Assay 2	Mean	Mean
Verbal learning and memory	HVLT-R				
Visual learning and memory	BVMT-R				
	RCFT- copy				
	RCFT - immediate recall				
	RCFT - delayed recall				
Working memory	CBTT				
	LNS		*		
Processing speed	TMT - A				
	BACS SC				
	Fluency				
	Stroop Direct W				
	Stroop Direct C				
Attention/vigilance	CPT-IP				
Executive function	TMT- B				
	NAB Mazes				
	Stroop Direct WC				
	Stroop Direct Interference				

*Statistically significant results ($p < 0.05$).

TMT-A and TMT-A r values are inverted (since higher results in these tests imply a worse cognitive function).

Abbreviations: HVLT-R, Hopkins Verbal Learning Test-Revised; BVMT-R, Brief Visuospatial Memory Test-Revised; RCFT, Rey Complex Figure Test; LNS, Letter Number Span; TMT-A, Trail Making Test part A; BACS-SC, Brief Assessment of Cognition in Schizophrenia-Symbol Coding; W, words; C, colors; CPT-IP, Continuous Performance Test-Identical Pairs; TMT-B, Trail Making Test part B; NAB-Mazes, Neuropsychological Assessment Battery-Mazes; WC, words-colors; HDRS, Hamilton Depression Rating Scale; STAI, State-Trait Anxiety Inventory; CTQ, Childhood Trauma Questionnaire; DDD, Defined Daily Dose.

r values
0.5 to 0.6
0.4 to 0.5
0.3 to 0.4
0.2 to 0.3
0.1 to 0.2
0 to 0.1
0 to -0.1
-0.1 to -0.2
-0.2 to -0.3
-0.3 to -0.4
-0.4 to -0.5
-0.5 to -0.6
