# Supplementary material for: BDNF genetic variants and methylation: effects on cognition in major depressive disorder
Source: Transl Psychiatry. 2019 Oct 21;9:265. doi: 10.1038/s41398-019-0601-8 (PMC6803763; doi:10.1038/s41398-019-0601-8)
Supplement: Supplementary file 6 — Table S3 [file 41398_2019_601_MOESM6_ESM.pdf]

Table S3.
Results of multiple linear regression analyses of methylation in promoter I (Assay 1) and neuropsychological performance in all participants

	Mean	CpG_1	CpG_6	CpG_7_8_9	CpG_10	CpG_11_12	CpG_15	CpG_26
	β	β	β	β	β	β	β	β
Verbal learning and memory								
HVLT-R	0.100	0.058	-0.078	-0.121	**0.145***	0.029	0.074	0.095
Visual learning and memory								
BVMT-R	-0.042	-0.014	-0.078	**-0.226****	**0.147***	-0.035	-0.119	-0.081
RCFT- copy	0.102	0.021	-0.051	-0.037	**0.227****	-0.110	0.081	0.014
RCFT - immediate recall	<0.001	0.023	-0.077	**-0.170***	**0.160***	-0.118	-0.029	-0.055
RCFT - delayed recall	-0.009	0.012	-0.095	**-0.153***	**0.153***	-0.122	-0.040	-0.049
Working memory								
CBTT	0.083	-0.033	0.022	-0.088	**0.200****	-0.043	0.007	0.069
LNS	-0.129	-0.127	-0.115	**-0.196****	0.057	-0.085	**-0.169***	-0.084
Processing speed								
TMT - A	-0.045	0.007	0.075	0.088	**-0.166***	-0.053	0.048	-0.010
BACS SC	-0.022	-0.106	-0.091	-0.105	**0.129***	0.011	-0.049	-0.077
Fluency	-0.029	-0.083	-0.084	**-0.233****	0.096	-0.017	-0.019	-0.020
Stroop Direct W	0.080	0.019	-0.103	-0.127	0.142	**0.192***	0.026	0.008
Stroop Direct C	0.059	-0.053	-0.076	**-0.197***	0.155	0.073	0.013	0.084
Attention/vigilance								
CPT-IP	0.069	0.016	0.036	**-0.198***	**0.224****	-0.014	-0.026	0.006
Executive function								
TMT- B	-0.053	-0.033	-0.097	**0.152***	-0.095	-0.003	<0.001	-0.045
NAB Mazes	0.026	-0.075	-0.110	**-0.146***	**0.193****	-0.013	-0.032	0.013
Stroop Direct WC	0.087	-0.026	-0.026	-0.107	0.136	0.071	0.021	0.132
Stroop Direct Interference	0.062	-0.014	0.052	0.015	0.045	-0.007	0.010	0.147

Statistically significant results are highlighted (* p<0.05; ** p<0.01; *** p<0.001)

Linear regression analyses adjusted by sex, age, years of education, MDD diagnosis, tobacco consumption, HDRS, STAI trait score and CTQ score.

Abbreviations: β, Standardized beta coefficient; HVLT-R, Hopkins Verbal Learning Test-Revised; BVMT-R, Brief Visuospatial Memory Test-Revised; RCFT, Rey Complex Figure Test; CBTT, Corsi Block-Tapping Test; LNS, Letter Number Span; TMT-A, Trail Making Test Part A; BACS-SC, Brief Assessment of Cognition in Schizophrenia-Symbol Coding; W, words; C, colors; CPT-IP, Continuous Performance Test-Identical Pairs; TMT-B, Trail Making Test Part B; NAB-Mazes, Neuropsychological Assessment Battery-Mazes; WC, words-colors.
