# Supplementary material for: BDNF genetic variants and methylation: effects on cognition in major depressive disorder
Source: Transl Psychiatry. 2019 Oct 21;9:265. doi: 10.1038/s41398-019-0601-8 (PMC6803763; doi:10.1038/s41398-019-0601-8)
Supplement: Supplementary file 7 — Table S4 [file 41398_2019_601_MOESM7_ESM.pdf]

Table S4.
Results of multiple linear regression analyses of methylation in promoter I (Assay 2) and neuropsychological performance in all participants

	Mean	CpG_1	CpG_2	CpG_3_4_5_6	CpG_9	CpG_11	CpG_12	CpG_14
	β	β	β	β	β	β	β	β
Verbal learning and memory								
HVLT-R	-0.030	0.021	-0.058	-0.073	0.062	0.011	0.024	-0.032
Visual learning and memory								
BVMT-R	-0.144	0.011	-0.095	-0.106	-0.077	**-0.163***	-0.106	-0.049
RCFT- copy	-0.047	-0.042	-0.032	-0.013	0.033	-0.010	<0.001	-0.074
RCFT - immediate recall	-0.108	-0.034	-0.082	-0.028	-0.114	-0.001	-0.101	-0.073
RCFT - delayed recall	-0.046	-0.019	-0.031	0.026	-0.077	0.019	-0.027	-0.073
Working memory								
CBTT	0.065	-0.058	0.011	0.065	-0.024	0.039	0.009	0.099
LNS	**-0.207****	-0.081	-0.128	**-0.144***	**-0.208****	-0.121	-0.087	-0.087
Processing speed								
TMT - A	0.031	0.054	-0.031	0.069	-0.089	-0.077	0.025	0.036
BACS SC	0.029	0.051	0.001	-0.047	0.027	0.068	0.071	0.007
Fluency	0.009	0.128	-0.067	-0.116	-0.021	-0.028	0.024	0.094
Stroop Direct W	-0.004	-0.024	0.152	-0.075	0.029	-0.055	0.098	-0.026
Stroop Direct C	0.029	0.065	0.028	-0.110	0.044	0.018	0.067	0.064
Attention/vigilance								
CPT-IP	-0.019	-0.099	-0.014	-0.019	-0.002	-0.083	0.046	0.021
Executive function								
TMT- B	-0.066	-0.097	-0.103	-0.011	0.053	-0.001	-0.073	-0.040
NAB Mazes	-0.019	0.001	-0.016	-0.006	0.032	0.058	0.027	-0.082
Stroop Direct WC	0.091	0.034	0.042	-0.014	0.060	0.072	0.073	0.096
Stroop Direct Interference	0.125	0.025	-0.012	0.078	0.055	0.109	0.029	0.123

Statistically significant results are highlighted (* p<0.05; ** p<0.01; *** p<0.001)

Linear regression analyses adjusted by sex, age, years of education, MDD diagnosis, tobacco consumption, HDRS, STAI trait score and CTQ score.

Abbreviations: β, Standardized beta coefficient; HVLT-R, Hopkins Verbal Learning Test-Revised; BVMT-R, Brief Visuospatial Memory Test-Revised; RCFT, Rey Complex Figure Test; CBTT, Corsi Block-Tapping Test; LNS, Letter Number Span; TMT-A, Trail Making Test Part A; BACS-SC, Brief Assessment of Cognition in Schizophrenia-Symbol Coding; W, words; C, colors; CPT-IP, Continuous Performance Test-Identical Pairs; TMT-B, Trail Making Test Part B; NAB-Mazes, Neuropsychological Assessment Battery-Mazes; WC, words-colors.
