# Supplementary material for: BDNF genetic variants and methylation: effects on cognition in major depressive disorder
Source: Transl Psychiatry. 2019 Oct 21;9:265. doi: 10.1038/s41398-019-0601-8 (PMC6803763; doi:10.1038/s41398-019-0601-8)
Supplement: Supplementary file 8 — Table S5 [file 41398_2019_601_MOESM8_ESM.pdf]

Table S5.

Results of multiple linear regression analyses of methylation in promoter IV and neuropsychological performance in all participants

	Mean β	CpG_3_4 β	CpG_5 β	CpG_6_7_8 β	CpG_9 β	CpG_10 β	CpG_11 β	CpG_13 β	CpG_15_16_17 β	CpG_20 β	CpG_21 β	CpG_22_23 β
Verbal learning and memory												
HVLT-R	-0.013	0.096	-0.029	-0.078	**-0.162***	-0.067	0.095	-0.026	0.109	0.003	0.072	0.069
Visual learning and memory												
BVMT-R	**-0.152***	-0.001	-0.091	-0.045	**-0.173***	-0.077	0.036	-0.116	-0.041	-0.100	0.010	-0.085
RCFT- copy	-0.111	0.058	-0.103	-0.067	-0.123	-0.158	**0.168***	**-0.191***	0.042	-0.105	0.047	0.052
RCFT - immediate recall	**-0.240****	-0.061	**-0.165***	-0.055	**-0.177***	**-0.162***	0.064	**-0.277*****	-0.028	-0.138	0.029	-0.069
RCFT - delayed recall	**-0.261*****	-0.057	**-0.148***	-0.053	**-.0207****	**-0.189***	0.035	**-0.313*****	-0.029	-0.144	0.020	-0.023
Working memory												
CBTT	-0.014	0.024	-0.023	0.128	-0.052	-0.118	0.076	**-0.177***	0.015	-0.072	0.081	0.062
LNS	**-0.177***	-0.060	-0.050	-0.085	**-0.171***	-0.111	-0.019	-0.014	-0.135	-0.039	-0.047	**-0.138***
Processing speed												
TMT - A	0.106	-0.025	0.034	0.070	0.120	**0.170***	-0.038	0.119	-0.073	0.103	-0.080	0.015
BACS SC	-0.048	-0.003	-0.065	-0.009	**-0.146**	-0.083	**0.109***	-0.079	0.070	-0.012	0.026	0.019
Fluency	**-0.175***	-0.013	**-0.168***	-0.074	**-0.157***	**-0.227****	0.013	-0.082	0.063	-0.100	-0.014	-0.038
Stroop Direct W	0.068	0.068	0.029	-0.034	-0.113	-0.107	0.124	0.030	**0.176***	0.077	0.071	0.073
Stroop Direct C	-0.029	0.076	-0.019	-0.032	-0.142	**-0.182***	0.036	-0.064	0.146	0.005	0.085	0.040
Attention/vigilance												
CPT-IP	-0.046	0.034	-0.069	-0.047	-0.136	-0.081	0.055	<0.001	-0.012	0.052	0.050	-0.045
Executive function												
TMT- B	0.061	-0.010	0.063	-0.056	**0.199****	0.107	-0.076	0.094	0.012	0.013	-0.116	0.066
NAB Mazes	-0.124	-0.046	-0.062	-0.041	-0.096	**-0.151***	0.040	-0.118	-0.092	-0.047	0.069	-0.023
Stroop Direct WC	<0.001	0.026	0.025	-0.041	-0.088	-0.037	0.013	-0.096	0.125	0.071	0.056	0.087
Stroop Direct Interference	-0.010	-0.031	0.042	-0.030	-0.003	0.101	-0.045	-0.121	0.034	0.062	-0.004	0.085

Statistically significant results are highlighted (* p<0.05; ** p<0.01; *** p<0.001)

Linear regression analyses adjusted by sex, age, years of education, MDD diagnosis, tobacco consumption, HDRS, STAI trait score and CTQ score.

Abbreviations: β, Standardized beta coefficient; HVLT-R, Hopkins Verbal Learning Test-Revised; BVMT-R, Brief Visuospatial Memory Test-Revised; RCFT, Rey Complex Figure Test; CBTT, Corsi Block-Tapping Test; LNS, Letter Number Span; TMT-A, Trail Making Test Part A; BACS-SC, Brief Assessment of Cognition in Schizophrenia-Symbol Coding; W, words; C, colors; CPT-IP, Continuous Performance Test-Identical Pairs; TMT-B, Trail Making Test Part B; NAB-Mazes, Neuropsychological Assessment Battery-Mazes; WC, words-colors.
