# Supplementary material for: BDNF genetic variants and methylation: effects on cognition in major depressive disorder
Source: Transl Psychiatry. 2019 Oct 21;9:265. doi: 10.1038/s41398-019-0601-8 (PMC6803763; doi:10.1038/s41398-019-0601-8)
Supplement: Supplementary file 9 — Table S6 [file 41398_2019_601_MOESM9_ESM.pdf]

Table S6.

Statistically significant results of multiple linear regression analyses exploring the interaction between methylation variables with sex, CTQ and MDD diagnosis in association to cognitive performance.

SEX

Promoter I

	Cognitive test	β CpG	β sex (female)	Interaction CpG x sex
Assay 1:				
Mean	Fluency	-0.225	-0.545	**0.590**
CpG_11_12	HVLT-R	-0.168	-0.406	**-0.168**
	Fluency	**-0.288**	**-0.608**	**0.727**
CpG_15	Stroop Direct W	-0.196	**-0.435**	**0.403**
CpG_26	Stroop Direct C	-0.174	**-0.458**	**0.571**
Assay 2:				
CpG_11	CBTT	0.306	0.491	**-0.850**

Promoter IV

	Cognitive test	β CpG	β sex (female)	Interaction CpG x sex
CpG_3_4	LNS	0.156	0.450	**-0.655**
CpG_6_7_8	Stroop Direct Interference	0.214	0.396	**-0.558**
	RCFT - delayed recall	0.142	0.251	**-0.444**
CpG_9	NAB Mazes	**-0.389**	**-0.615**	**0.465**
CpG_22_23	TMT_B	**0.256**	**0.404**	**-0.400**
	Stroop Direct W	**-0.262**	**-0.689**	**0.698**
	Stroop Direct C	**-0.289**	**-0.521**	**0.686**
	BACS SC	-0.120	-0.218	**0.300**
	HVLT-R	-0.188	**-0.638**	**0.530**
	NAB Mazes	**-0.236**	**-0.602**	**0.451**
	CPT IP	**-0.275**	**-0.441**	**0.512**

CTQ

Promoter I

	Cognitive test	β CpG	β CTQ	Interaction CpG x CTQ
Assay 1:				
CpG_6	HVLT-R	**2.972**	**0.563**	**-3.001**
CpG_11_12	CBTT	**2.625**	**0.569**	**-2.823**
	CPT-IP	2.026	0.472	**-2.179**
Assay 2:				
Mean	TMT - A	**-2.432**	**-0.979**	**2.632**
CpG_9	Stroop Direct C	**2.787**	**0.421**	**-2.806**
CpG_11	TMT - A	**-1.757**	-0.560	**1.829**
CpG_12	TMT - A	**-2.505**	2.546	**-0.247**
CpG_14	CPT-IP	**2.272**	**0.652**	**-2.299**

Promoter IV

	Cognitive test	β CpG	β CTQ	Interaction CpG x CTQ
Mean	TMT - A	**-1.976**	**-1.267**	**2.495**
CpG_3_4	Stroop Direct Interference	**-2.322**	**-0.827**	**2.379**
CpG_11	RCFT - immediate recall	**2.618**	**1.497**	**-2.970**
	RCFT - delayed recall	**2.533**	**1.492**	**-2.905**
	TMT - A	**-2.414**	**-1.360**	**2.763**
CpG_13	TMT - A	**-2.523**	**-0.644**	**2.730**
CpG15_16_17	RCFT - immediate recall	**2.259**	**1.159**	**-2.298**

MDD Diagnosis

Promoter I

	Cognitive test	β CpG	β Diagnosis	Interaction CpG x MDD
Assay 1:				
CpG_6	Stroop Direct W	0.026	0.553	**-0.543**
	RCFT - immediate recall	0.078	0.456	**-0.647**
	RCFT - delayed recall	0.047	0.348	**-0.589**
	Fluency	0.037	0.456	**-0.508**
CpG_10	Fluency	**0.281**	**0.341**	**-0.480**
CpG_11_12	RCFT - immediate recall	0.075	0.414	**-0.633**
	RCFT - delayed recall	0.044	0.281	**-0.548**
CpG_26	TMT B	**-0.196**	-0.241	**0.422**
	RCFT - immediate recall	0.108	0.253	**-0.456**
	LNS	0.073	0.104	**-0.445**
	NAB Mazes	0.171	0.226	**-0.445**
	Fluency	0.133	0.362	**-0.430**
Assay 2:				
CpG_1	Stroop Direct W	0.191	0.201	**-0.353**
	Stroop Direct C	0.210	-0.008	**-0.238**
	Stroop Direct WC	0.178	-0.053	**-0.235**
	RCFT - copy	0.157	-0.046	**-0.328**
	Fluency	**0.302**	0.140	**-0.288**
	BACS SC	**0.154**	-0.060	**-0.170**
CpG_9	CBTT	0.133	0.064	**-0.366**
	Stroop Direct W	0.191	0.334	**-0.384**
CpG_12	CPT-IP	-0.073	**-0.429**	**0.295**

Promoter IV

	Cognitive test	β CpG	β Diagnosis	Interaction CpG x MDD
CpG_3_4	Stroop Direct Interference	-0.208	**-0.927**	**0.747**
	RCFT - delayed recall	0.096	0.436	**-0.643**
CpG_5	TMT B	-0.067	-0.354	**0.534**
	BVMT-R	0.095	**0.456**	**-0.737**
CpG_6_7_8	HVLT-R	0.049	0.335	**-0.444**
CpG_9	CBTT	0.128	0.032	**-0.365**
	Stroop Direct W	0.095	0.336	**-0.436**
	CPT-IP	0.089	0.111	**-0.472**
CpG_10	RCFT - copy	0.004	0.016	**-0.377**
CpG_13	TMT B	-0.049	-0.300	**0.480**
	LNS	0.133	0.163	**-0.482**
	BVMT-R	0.026	0.181	**-0.447**
CpG_20	RCFT - copy	0.022	-0.005	**-0.309**

Statistically significant results highlighted.

All linear regressions adjusted by sex, age, years of education, tobacco consumption, HDRS, STAI trait score and CTQ score. Cognitive tests were considered the dependent variable for each multiple linear regression analysis.

Abbreviations: β, Standardized beta coefficient; HVLT-R, Hopkins Verbal Learning Test-Revised; BVMT-R, Brief Visuospatial Memory Test-Revised; RCFT, Rey Complex Figure Test; CBTT, Corsi Block-Tapping Test; LNS, Letter Number Span; TMT-A, Trail Making Test part A; BACS-SC, Brief Assessment of Cognition in Schizophrenia-Symbol Coding; W, words; C, colors; CPT-IP, Continuous Performance Test-Identical Pairs; TMT-B, Trail Making Test part B; NAB-Mazes, Neuropsychological Assessment Battery-Mazes; WC, words-colors.
